# Polymorphism of Antifolate Drug Resistance in *Plasmodium vivax* From Local Residents and Migrant Workers Returned From the China-Myanmar Border

**DOI:** 10.3389/fcimb.2021.683423

**Published:** 2021-06-24

**Authors:** Weilin Zeng, Siqi Wang, Shi Feng, Daibin Zhong, Yue Hu, Yao Bai, Yonghua Ruan, Yu Si, Hui Zhao, Qi Yang, Xinxin Li, Xi Chen, Yanmei Zhang, Cuiying Li, Zheng Xiang, Yanrui Wu, Fang Chen, Pincan Su, Benjamin M. Rosenthal, Zhaoqing Yang

**Affiliations:** ^1^ Department of Pathogen Biology and Immunology, Kunming Medical University, Kunming, China; ^2^ Program in Public Health, College of Health Sciences, University of California at Irvine, Irvine, CA, United States; ^3^ Department of Pathology, Kunming Medical University, Kunming, China; ^4^ Department of Cell Biology and Medical Genetics, Kunming Medical University, Kunming, China; ^5^ Transfusion Medicine Research Department, Yunnan Kunming Blood Center, Kunming, China; ^6^ Animal Parasitic Disease Laboratory, Agricultural Research Service, US Department of Agriculture, Beltsville, MD, United States

**Keywords:** *Plasmodium vivax*, sulfadoxine-pyrimethamine, dihydropteroate synthase gene, dihydrofolate reductase gene, GTP cyclohydrolase I gene

## Abstract

Drug-resistant *Plasmodium vivax* malaria impedes efforts to control, eliminate, and ultimately eradicate malaria in Southeast Asia. *P. vivax* resistance to antifolate drugs derives from point mutations in specific parasite genes, including the dihydropteroate synthase (*pvdhps*), dihydrofolate reductase (*pvdhfr*), and GTP cyclohydrolase I (*pvgch1*) genes. This study aims to investigate the prevalence and spread of drug resistance markers in *P. vivax* populating the China-Myanmar border. Blood samples were collected from symptomatic patients with acute *P. vivax* infection. Samples with single-clone *P. vivax* infections were sequenced for *pvdhps* and* pvdhfr* genes* *and genotyped for 6 flanking microsatellite markers. Copy number variation in the *pvgch1* gene was also examined. Polymorphisms were observed in six different codons of the *pvdhps* gene (382, 383, 512, 549, 553, and 571) and six different codons of the *pvdhfr* gene (13, 57, 58, 61, 99, 117) in two study sites. The quadruple mutant haplotypes 57I/L/58R/61M/117T of *pvdhfr* gene were the most common (comprising 76% of cases in Myitsone and 43.7% of case in Laiza). The double mutant haplotype 383G/553G of *pvdhps* gene was also prevalent at each site (40.8% and 31%). Microsatellites flanking the *pvdhfr* gene differentiated clinical samples from wild type and quadruple mutant genotypes (*F*_ST_= 0.259-0.3036), as would be expected for a locus undergoing positive selection. The lack of copy number variation of *pvgch1 *suggests that SP-resistant *P. vivax *may harbor alternative mechanisms to secure sufficient folate.

## Introduction

*Plasmodium vivax*, the most broadly distributed source of severe malarial morbidity and mortality, puts almost 40% of the world population at risk (Naing et al., 2014; Rahimi et al., 2014; WHO, 2020), resulting in cases that can be life threatening (Gupta et al., 2015; Gupta et al., 2016). Data from 2017 shows that about 3.3 billion people live in the *P. vivax* transmission areas, and about 1.5 billion people are at risk of exposure in foci of stable transmission (Battle et al., 2019). Southeast Asian countries shoulder the highest proportion of global burden, with about 7.3 million people infected with *P. vivax* in 2017, alone (Battle et al., 2019).

Asia is the focus of *P. vivax* transmission, and Myanmar is among the most severely affected in the region. As one of the most severe malaria burden countries from this area, Myanmar is a source of cases exported to neighboring countries such as mainland China, where low endemic transmission is otherwise leading to successful eradication. From 2010 to 2014, there were 1893 P*. vivax* malaria cases imported from Myanmar into China, far exceeding importation from any other Asian country (Zhou et al., 2016). Most imported cases are adult male Chinese laborers working in farming, construction, and mining (Zhou et al., 2016). Reported cases in Myanmar have risen steadily since 2012 (WHO, 2016).

Most cases imported to China occur in Yunnan province, the southwestern frontier which borders Myanmar to the west, and Laos and Vietnam to the south (Lai et al., 2019). In particular, Tengchong County (in Yunnan, China) reported the highest number of imported malaria cases from Myitsone, Myanmar from 2010 to 2014 (Wang et al., 2015). Previous studies identified boundary townships, where roads span the China-Myanmar Border, facilitate the spread of *P. vivax* (Hu et al., 2016; Xu et al., 2016).Therefore, the area merits significance for global malaria control.

The recommended first-line medication for vivax malaria patients in Myanmar is a radical cure with chloroquine-primaquine (CQ-PQ) (Htun et al., 2017). However, most local cases of malaria derive from *Plasmodium falciparum *(or from mixed infections with *P. falciparum and P. vivax*). Although the prevalence of mixed-species malaria infections was previously estimated at <2% in some surveys of Asian locales, therapeutic studies identify coinfection in over 30% of cases (Mayxay et al., 2004). The incidence of *P. vivax* infection after *falciparum* malaria treatment exceeds that which would be expected from entomological inoculation rates in Southeast Asia (Douglas et al., 2011). A survey from 2008 to 2012 in Yunnan found 17% of malaria cases were co-infections with *P. vivax* and *P. falciparum*, underscoring the difficulty in diagnosing low-level and co-infection cases (Zhou et al., 2014).

Given such high rates of coinfection, treatments meant to target chloroquine-resistant *P. falciparum* likely impose substantial selective pressure for resistance to sulfadoxine-pyrimethamine (SP) in the local *P. vivax *population, and may induce high-grade antifolate resistance in *P. vivax*. SP has been the recommended treatment for *falciparum* and other non-*vivax* malaria since the 1980s, replacing chloroquine (CQ) as the front-line anti-malaria treatment when large-scale CQ resistance developed in many countries; but growing drug resistance soon necessitated replacement of SP with artemisinin-based combination therapy (ACT) (Jiang et al., 2020). In addition to typical treatment proscribed by WHO guidelines (WHO, 2013), SP is also used for intermittent preventive treatment in infants (IPTi) and pregnant women (IPTp) in areas endemic for malaria.

Meanwhile, China developed its own strategy of malaria control: seasonal mass drug administration with pyrimethamine and primaquine. This approach attempts to treat carriers of *P. vivax* before seasonal transmission begins (Hsiang et al., 2013). These several uses of such antimalarials near the China-Myanmar Border necessitate continuous monitoring of antifolate resistance in *P. vivax* in this area. Given growing concerns of drug resistance, it is essential to monitor antifolate resistance in *P. vivax* in this area, which has undergone changes in transmission intensity and drug therapy.

Antifolate resistance derives, in part, from point mutations of enzymes used by the parasite to synthesize folate. These include dihydropteroate synthase (DHPS) and dihydrofolate reductase (DHFR). Barriers to continuous culture of *P. vivax* severely limit efforts to link *pvdhfr* and *pvdhps* genotypes with parasite responses to SP *in vitro*. Instead, investigators assay drug sensitivity by expressing *P. vivax* DHPS and DHFR in *E. coli*or yeast (Auliff et al., 2010). In *P. falciparum*, SP resistance has also been attributed to amplification of *GTP-cyclohydrolase* (*gch1*), the first gene in the folate biosynthesis pathway (Nair et al., 2008); this phenomenon has not yet been demonstrated in *P. vivax.* Drug resistance was last surveyed from the vicinity (Nabang Town, Yingjiang County, Yunnan Province, and Kachin, Myanmar were surveyed in 2015 and 2017) (Wang et al., 2020; Zhao et al., 2020); sampling from Myitsone has not yet been reported.

Despite their likely contribution to emerging regional drug resistance, the presence and prevalence of mutations in genes of the folate biosynthesis pathways in *P. vivax* remain uncharacterized. This study aims to investigate the extent and spread of SP drug resistance alleles in *P. vivax* from local people in Laiza, Myanmar and from migrant Chinese laborers returning from Myitsone, Myanmar between 2012 and 2015.To do so, we sought signatures of selection by sequencing SP resistance-associated molecular markers and genotyped microsatellite markers around target genes.

## Material and Methods

### Samples

**Figure 1:**
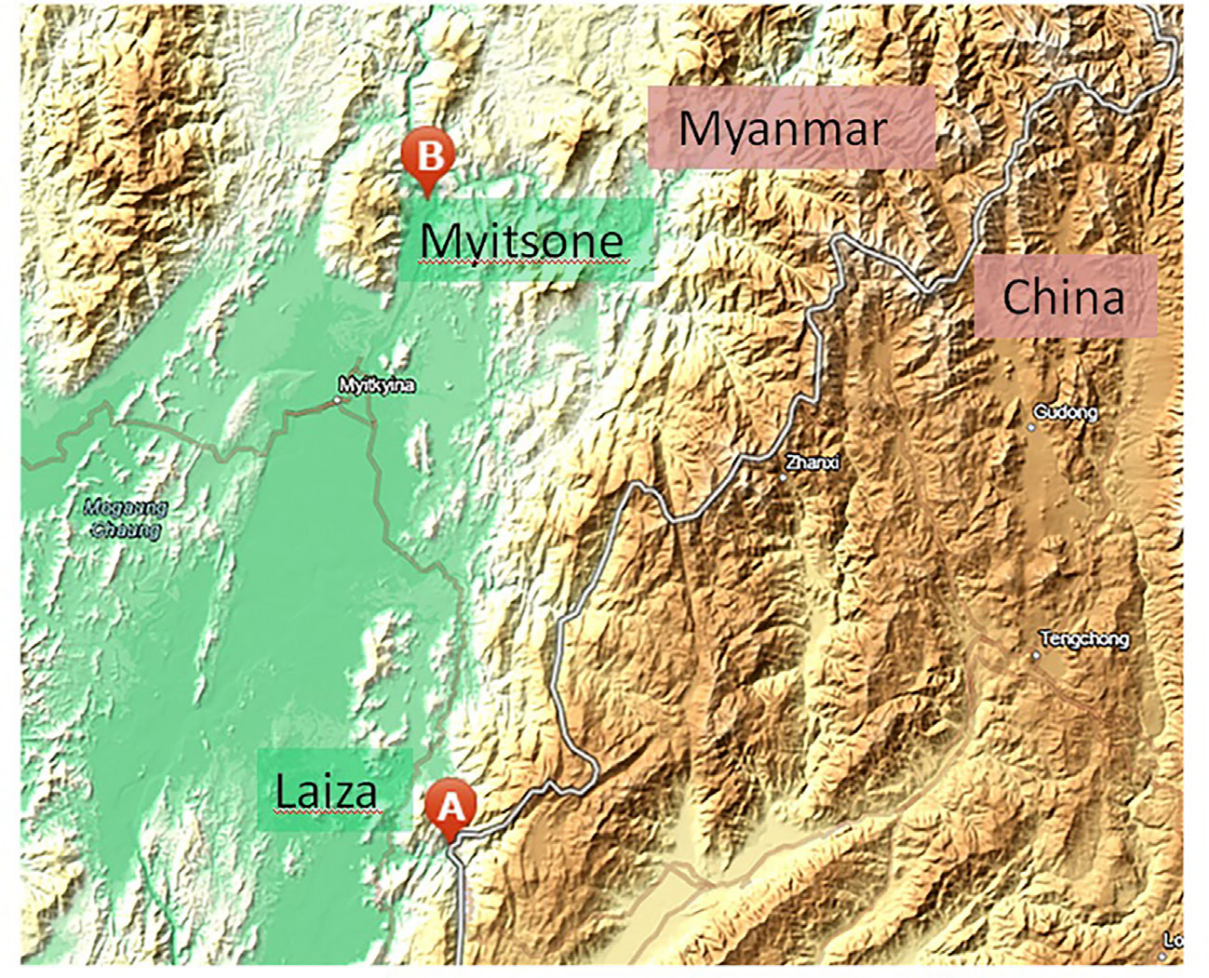
Map of sampling sites.

Between 2012 and 2015, finger-prick blood samples were collected from symptomatic patients with acute *P. vivax* infection. Infections were diagnosed by microscopy using Giemsa-stained thick and thin blood films at 13 local clinics around the Laiza township (N 24° 45’ 24”, E 97° 33’ 02”), Myanmar, and in returning migrant Chinese laborers from the Myitsone area (N 25° 41’ 23”, E 97° 31’ 04”), Myanmar (**Figure 1**). Patients who provided blood were well-informed by our study team and signed a consent form before sampling. The Institutional Review Board of Kunming Medical University, China, and the local Bureau of Health, Kachin State, Myanmar approved the sampling procedures.

Parasite DNA was extracted from each sample using the High Pure PCR Template Preparation Kit (Roche, Germany). Multiclonal infections were identified by genotyping the single-copy antigenic genes *msp-3α *and *msp-3β *as described previously (Khan et al., 2014); these were excluded from the ensuing analysis, leaving a total of 308 single-clone *P. vivax* isolates (101 from Myitsone, 207 from Laiza). A sample of fewer than 100 would suffice to achieve 80% power (with a type I error <.05) given reported mutation frequencies of in *pvdhfr* and *pvdhps.*

### *Pvdhfr* and *pvdhps* Gene Sequencing, *Pvgch1* Copy Number Quantification, and Microsatellite Genotyping

Parasite genes *pvdhfr* and *pvdhps* were amplified by nested PCR using Premix Taq Version 2.0 plus dye (TaKaRa Bio, Japan), according to the manufacturer’s instructions. Primers and PCR conditions were as described in **Supplementary Table S1**. PCR products were purified and sequenced by Sanger at SinoGenoMax (Kunming, China). For accuracy, sequences from the forward and reverse were examined by alignment using DNASTAR software (v.7.1). To avoid technical contamination, all PCR and sequencing assays include positive control (MRA-41 Sal I, gDNA, MR4/Bei resources) and negative controls (uninfected samples and water). Mutations were scored only when confirmed by each bidirectional sequencing read with high quality BioEdit (v.7.2.5) (Sarmah et al., 2017) was used to compare individual sequences with the reference sequences *pvdhfr* (PVX_089950) and *pvdhps* (PVX_123230). Nucleotide sequences were translated into amino acid sequences to examine mutant codons.

To determine copy number variation in *pvgch1*, PCR primers were designed (**Supplementary Table S1**) and quantitative real-time (qRT-PCR) performed using SuperRealPreMix Plus (SYBR Green) (TIANGEN, China) on the Stratagene Mx3000P PCR machine (Stratagene, CA, USA), according to the manufacturer’s instructions. The threshold cycle value (Ct value) of *pvgch1* was estimated using *pvaldolase* the reference gene. In particular, we assessed, *via* real-time PCR, the proportion of mutant alleles of *pvgch1* compared with the reference allele. This was calculated using Pfaffl method for each patient sample as amplification efficiency of the target and reference was not similar. Results were recorded as a ratio calculated from the mean Ct values for each gene in each sample. The pUC57 plasmid inserted with a single copy of *pvgch1* or *pvaldolase* was purchased from Sangon Biotech (Shanghai, China) and used for amplification efficiency calibration.

Microsatellites flanking the *pvdhfr* gene were genotyped as previously reported (Ding et al., 2013). In brief, we analysed six microsatellite loci, with more than eight repeats, located proximate to the *dhfr* gene (-93 kb, -38 kb, -2.6 kb, +4.9 kb, +37 kb, and +94 kb) on chromosome 5 (GI: 157098056). Products were amplified by PCR using Premix TaqVersion 2.0 plus dye (TaKaRa Bio, Japan). Products labelled with Hex fluorophore were separated and detected on ABI 3730 capillary sequencer (Thermo Fisher Scientific, US) by Sangon Biotech (Shanghai, China). DNA fragment sizes were determined and visualized using Gene Marker software (v.1.5.).

### Data Analysis

Population genetic parameters, pairwise linkage disequilibrium (LD), and tests of neutrality were estimated using DNASP software (v.5.0.) (Mardani et al., 2012). The copy number of *pvgch1* in each sample was estimated by the Pfaffl method using the Ct value, rounded to the nearest integer (Raju et al., 2019).

The Chi-square test and Fisher’s exact test were employed to compare the independence of categorical variables, using GraphPad Prism 9.0.0 software. A statistical significance was set at the *P*-value of < 0.05. The length polymorphism of microsatellite loci and allele frequencies were calculated using GenAlEx software (v.6.5.) (He et al., 2018). Expected heterozygosity (*H_e_*) was estimated for each locus based on allele frequencies following previously reported methods (Anderson et al., 2000). The sampling variance for *H_e_*was estimated using ARLEQUIN software (v.3.5.), allowing estimation of genetic diversity indices and assessment of population differentiation between haplotypes. The Bottleneck program (Cornuet and Luikart, 1996) was used to investigate the influence of selection on allele distributions under the infinite alleles (IMM) and step wise mutation models (SMM). Wilcoxon tests were used to determine the significance of departures from *H_e_*.

## Results

### Genetic Diversity of *pvdhfr* and *pvdhps* and Copy Number of *pvgch1*

A total 237 (76.9%) samples for *pvdhfr* (86 of Laiza and 151 of Myitsone) and 240 (77.9%) samples for *pvdhps* (98 of Laiza and142 of Myitsone)* *were successfully sequenced and analysed (**Table 1**). The amplification products of *pvdhfr* and *pvdhps* were 632 bp (PVX_089950, nucleotide positions at -38 to 5941-767, amino acids positions at 1-198)) and 767 bp (PVX_123230, nucleotide positions at 1380-2146, amino acid positions at 349-604), respectively. No length polymorphism was detected in either gene. More variant sites haplotypes were observed in *pvdhps* (14, 16) than *pvdhfr* (10,14).

**Table 1:** Genetic diversity and tests of neutrality of the *pvdhps* and *pvdhfr* genes in *P. vivax* samples from the China/Myanmar border.

Regions	N	S	H	Hd ± SD	π	Fu Li's F	Tajima's D
*dhfr* gene							
Myitsone	86	9	9	0.704 ± 0.033	0.003381	-0.66376	0.035381
Laiza	151	9	10	0.752 ± 0.020	0.006496	1.54068	2.75006**
Overall	237	10	14	0.784 ± 0.013	0.005948	0.72826	2.127615*
*dhps* gene							
Myitsone	98	7	10	0.784 ± 0.030	0.002263	-0.31995	0.275001
Laiza	142	11	11	0.759 ± 0.016	0.002037	-0.33432	-0.781062
Overall	240	14	16	0.783 ± 0.015	0.002240	-1.21122	-0.863855

N, Number of isolates; S, Number of segregating sites; H, Number of Haplotypes; Hd, haplotypes diversity; SD, Standard Deviation; π, average number of pairwise nucleotide diversity; *P < 0.05; **P < 0.01.

Nucleotide diversity in *pvdhfr* was almost twice as great in Laiza than in Myitsone (0.006496 *v.s.*0.003381); diversity was lower, and more regionally uniform, in *pvdhps* (0.002263 *v.s.*0.002037). For the Laiza population, neutrality tests showed significant positive values for Tajima’s D in the *pvdhfr* gene, indicating a dearth of singletons and consistent with balancing selection. However, for the *pvdhps* gene, we observed negative values for Fu & Li’s F and Tajima’s D, which indicates an excess of low-frequency alleles more consistent with population expansions or positive selection.

In the *pvdhfr* gene, Myitsone samples and Laiza samples contained mutations at six codons (13, 57, 58, 61, 99, 117)(**Table 2**). Compared to the *pvdhfr* reference sequence in Salvador I (PVX_089950), more than ninety percent of samples from each sampling locale had at least one codon change. Mutations in codons F57I/L, S58R, T61M, and S117T/N were highly prevalent in both populations. In the Myitsone samples, the F57I/L, S58R, T61M and S117T/N mutation in *pvdhfr* was significantly more frequent than in the Laiza samples; the H99R/S mutation in the Laiza samples was significantly more frequent than in the Myitsone samples. The quadruple mutant L_57_R_58_M_61_T_117_was observed significantly more frequent in Myitsone (44.2%) than in Laiza (12.6%) (**Table 3**). The quadruple mutant I_57_R_58_M_61_T_117_was observed in both areas (32.6% in Myitsone and 31.1% in Laiza). Genotypes R_99_ and S_99_ were only in Laiza (37.8%) (**Table 2**). Rare genotypes with triple mutations I_57_R_58_M_61_ (5 cases, 3.3%), R_58_R_99_T_117_ (1 case, 0.7%), and R_58_S_99_T_117_ (1 case, 0.7%) were also found in Laiza. The double mutant allele R_58_N_117_ existed in both areas, with prevalence of 5.8% and 4.6% in Myitsone and Laiza, respectively (**Table 3**).

**Table 2:** Prevalence distribution of *pvdhfr* and *pvdhps* gene mutations among two sites.

Gene	Mutation	Myitsone	Laiza	*P* value*
*pvdhfr*		n=86	n=151	
	I13L	2.3 (2)	1.3 (2)	0.6225
	F57I/L	80.2 (69)	48.3 (73)	**<0.0001**
	S58R	86.0 (74)	54.3 (82)	**<0.0001**
	T61M	79.1 (68)	48.3 (73)	**<0.0001**
	H99R/S	1.2 (1)	37.7 (57)	**<0.0001**
	S117T/N	90.7 (78)	51.0 (77)	**<0.0001**
*pvdhps*		n=98	n=142	
	S382A/C	20.4 (20)	4.9 (7)	**0.0003**
	A383G	84.7 (83)	73.9 (105)	0.0559
	K512E/M/T	5.1 (5)	2.1 (3)	0.2770
	G549D	1.0 (1)	0.0 (0)	0.4083
	A553G	65.3 (64)	38.0 (54)	**<0.0001**
	E571Q	0.0 (0)	6.3 (9)	**0.0118**

*Values shown in boldface were statistically significant (Fisher’s exact test).

**Table 3:** Prevalence of *pvdhfr* and *pvdhps* haplotypes in two sites

Gene and positions	Haplotype	Myitsone % (n)	Laiza % (n)	P*
***pvdhfr* (13, 57, 58, 61, 99, 117)**		N=86	N=151	
	IFSTHS(WT)	8.1 (7)	9.3 (14)	0.8174
	IFSTH**N**	4.7 (4)	–	**0.0166**
	IFST**S**S	–	36.4 (55)	**<0.0001**
	IFST**SN**	1.2 (1)	–	0.3629
	IF**R**TH**N**	5.8 (5)	4.6(7)	0.7613
	I**LR**THS	1.2 (1)	–	0.3629
	I**IRM**HS	–	3.3 (5)	0.162
	IF**R**T**RT**	–	0.7 (1)	1
	IF**R**T**ST**	–	0.7 (1)	1
	I**LRM**H**T**	44.2 (38)	12.6 (19)	**<0.0001**
	I**IRM**H**T**	32.6 (28)	31.1 (47)	0.8847
	**LLRM**H**T**	2.3 (2)	1.3 (2)	0.6225
***pvdhps* (382, 383, 512, 549, 553, 571)**		N=98	N=142	
	SAKGAE(WT)	9.2 (9)	24.6(35)	**0.0022**
	S**G**KGAE	17.3 (17)	31.0(44)	**0.0232**
	SAKG**G**E	6.1 (6)	1.4(2)	0.066
	**AG**KGAE	8.2 (8)	–	**0.0006**
	S**G**KG**G**E	40.8 (40)	31.0(44)	0.1309
	S**G**KGA**Q**	–	6.3 (9)	**0.0118**
	**AG**KG**G**E	12.2 (12)	2.1 (3)	**0.0021**
	S**GM**G**G**E	2.0 (2)	–	0.1657
	S**GT**G**G**E	3.1 (3)	–	0.0668
	S**G**K**DG**E	1.0 (1)	–	0.4083
	S**GE**G**G**E	–	0.7 (1)	1
	**CGE**G**G**E	–	2.8 (4)	0.1472

*Values shown in boldface were statistically significant (Fisher’s exact test).

For the pvdhps gene, nucleotide polymorphisms were observed at five different codons (382, 383, 512, 549, and 553) in samples from Myitsone and five codons (382, 383, 512, 553, and 571) from Laiza. As compared to the reference dhps sequence in Salvador I (PVX_123230), more than ninety percent and seventy percent of the samples had at least one codon change in Myitsone and Laiza samples, respectively. The most prevalent mutations were at codon A383G and A553G (**Table 2**). Similarly, these two codons also were more prevalent in the Myitsone population (84.7 and 65.3%, respectively) than in the Laiza population (74 and 38.1%, respectively). Mutation S382A also reached a high frequency (20.4%) in the Myitsone population. In the Myitsone samples the S382A/C and A553G mutation in pvdhps was significantly more frequent than in the Laiza samples; the E571Q mutation in the Laiza samples was significantly more frequent than in the Myitsone samples.

The parasites in Mytsome and Laiza each possessed certain unique *pvdhps* haplotypes (**Table 3**). For instance, parasites carrying the double mutations G_383_Q_571_ were identified only in Laiza, where they reached a sample prevalence of 6.3%. The double mutation A_382_G_383_ in *pvdhps* were 8.2% in Myitsone but not found in Laiza. Also, genotypes with triple mutations G_383_M_512_G_553_ (in 2 cases, 2%), G_383_T_512_G_553_ (in 3 cases, 3.1%), and G_383_D_549_G_553_ (in 1 case, 1%) were only found in Myitsone. However, the genotype with G_383_E_512_G_553_ were only found in 1 case (0.7%) in Laiza. Multiple mutations 382C/383G/512E/553G were found in 4 cases (3%) from Laiza. The distribution of major haplotypes of the *pvdhfr* and *pvdhps* genes in *P. vivax* from the two study areas are listed in **Table 3**.

Pairwise LD analysis revealed significant associations between several different nucleotide sites (as shown in **Supplementary Tables S2**, **S3**). The proportion of locus pairs showing significant LD was twice as high in Laiza (53.6%, 15/18 for *pvdhfr*; 50.9%, 28/55 for *pvdhfr*) than in Myitsone (25.0%, 7/28 for *pvdhfr*; 26.7%, 4/15 for *pvdhfr*). Only 7 of 101 samples in Myitsone had two copies of *gch1*; only 2 out of 101 had two *gch1* copies in Laiza (**Supplement Figure S1**). There was no significant difference in the two regions (*P* >0.05, Fisher’s exact test).

### Genetic Hitchhiking of Microsatellites Flanking the *pvdhfr* Gene

Polymorphisms of six microsatellite loci flanking *pvdhfr* (located at -93 kb, -38 kb, -2.6 kb, +4.9 kb, +37 kb, and +94 kb from the *pvdhfr* gene on chromosome 5) were examined in 100 samples from Laiza and 101 from Myitsone. Genotypes were obtained from 81 to 99 of the Myitone samples at these six loci, and from 65 to 97 of the Laiza samples (**Table 4**).

**Table 4:** Genetic diversity measured by microsatellites in the two population of *P. vivax* in Myanmar.

Microsatellite Locus	Myitsone			Laiza		
	N	Na	H_E_	N	Na	H_E_
-93 kb	92	6	0.784	95	9	0.696
-38 kb	81	6	0.644	65	8	0.714
-2.6 kb	98	9	0.814	97	15	0.742
+4.9 kb	99	5	0.702	96	7	0.418
+37 kb	92	3	0.479	90	6	0.594
+94 kb	96	9	0.712	94	16	0.894

N, sample size; Na, number of alleles; H_E_, expected heterozygosity.

**Figure 2:**
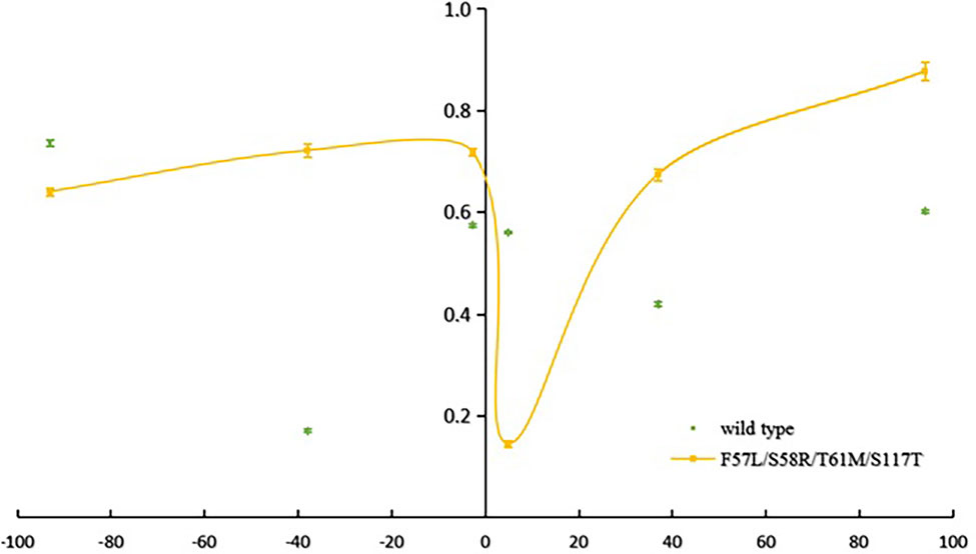
Reduced haplotype diversity proximate to mutant *pvdhfr* alleles in parasites sampled from Lazia (He, compared to wild type).

**Figure 3:**
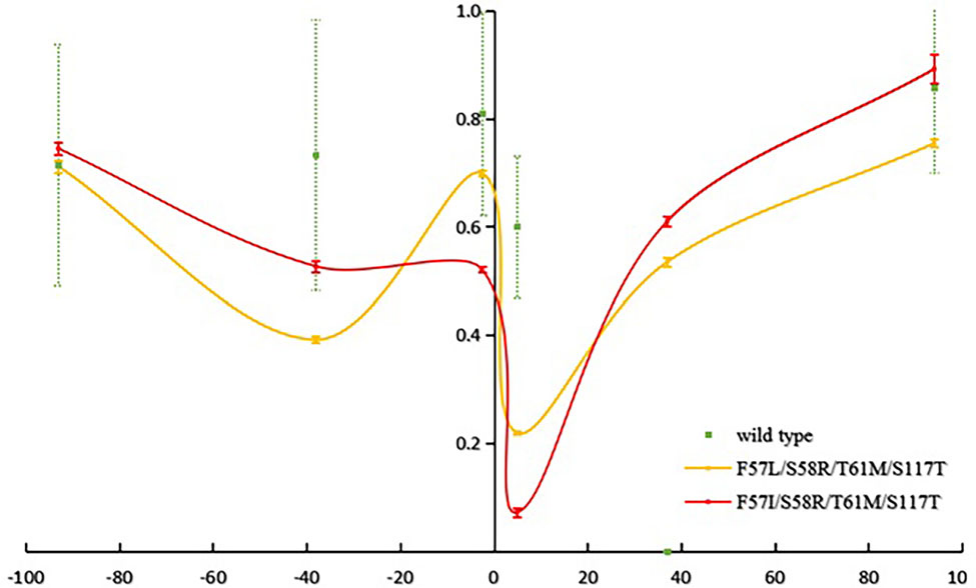
Reduced haplotype diversity proximate to mutant *pvdhfr* alleles in parasites sample from Myitsone (He, compared to wild type).

The mean number of observed alleles was higher in Laiza (10.2 ± 4.2) than in Myitsone (6.3 ± 2.3). A significant reduction in variability (compared to wild-type isolates) occurred across at the +4.9 kb region in Laiza samples and over a 7.5 kb region (spanning -2.6 to +4.9 kb) in Myitsone samples (*P*<0.01, Tukey’s multiple comparisons test) (**Figures 2** and **3**).

Tests using Bottleneck software, assuming the stepwise mutation model (SMM), indicated that parasites with the haplotype 57I/L-58R-61M-117T in the Myitsone population had significant heterozygote deficiency (*P*<0.05), suggestive of positive selection (**Supplementary Table S4**). However, significant heterozygosity excess (*P*<0.05) was found in parasites with genotype S58R-S117N in the Myitsone population, indicating that they could be suffering a bottleneck. There was no bottleneck effect detected in parasites with wild-type genotypes in either population when either an infinite allele (IMM) or stepwise mutation model (SMM) was assumed, suggesting parasite population under mutation-drift equilibrium. Significant genetic differentiation was observed between the wild type and the quadruple mutant genotypes 57I/L/58R/61M/117T (*F*_ST_=0.25492 for 57I/L/58R/61M/117T, *P*<0.00, *F*_ST_=0.30369 for 57I/L/58R/61M/117T, *P*<0.001) (as shown in **Table 5**).

**Table 5:** *F*_ST_ values for allele comparisons between different genotypes, determined using microsatellites around *dhfr* of *P. vivax* population in two regions of Myanmar.

Type of Allele	Myitsone			Laiza	
	Wild type	57L/58R/61M/117T	57I/58R/61M/117T	Wild type	57L/58R/61M/117T
Wild type					
57L/58R/61M/117T	0.2549*			0.4514*	
57I/58R/61M/117T	0.3036*	0.01163			

*P < 0.05.

## Discussion

An increasing number of drug resistance markers in *P. vivax *have spread throughout Southeast Asia (Lu et al., 2010; Thongdee et al., 2013; Asih et al., 2015). These include polymorphisms at codon 373, 380, 382, 383, 384, 512, 553, 585, and 601 in the *pvdhps* gene, which are associated with resistance to sulfadoxine, and mutations at codon 15, 33, 50, 57, 58, 61, 64, 117, and 173 in the *pvdhfr* gene, contributing to treatment failure using pyrimethamine. We found polymorphisms in five different codons of the *pvdhfr* gene (13, 57, 58, 61, 117) in each locale; mutations in a sixth codon (99) were restricted to Laiza. These locales each had polymorphisms in codons 382, 383, 512, 553 of the *pvdhps* gene; observed polymorphisms in codon 549 were restricted to Myitsone, and in codon 571 were restricted to Laiza. These results were consistent with those reported previously in Southeast Asia (Lu et al., 2010; Thongdee et al., 2013; Asih et al., 2015).

Though most of *the pvdhfr* and *pvdhps* haplotypes identified in this study have been reported in previous studies, some new haplotypes were also detected. For example, 2 cases each from Laiza and Myitsone bore quintuple-mutant 13L/57I/58R/61M/117T *pvdhfr* allele. To the best of our knowledge, this is the first report of this haplotype in the China-Myanmar border region. The S117N mutation has been suggested to represent the first mutational step conferring drug-resistance (Brega et al., 2004). However, as shown in the **Table 6**, only 5 cases in Myitsone bore the single S117N mutation, and the quadruple *pvdhfr* 57L/58R/61M/117T allele was the most prevalent; this differs from Yangon, Myanmar, where the double 58R/117N allele was most common (Lu et al., 2010).

**Table 6:** Distribution of major haplotypes of the *pvdhfr* and *pvdhps* genes in *P. vivax* from different regions in Myanmar and Yunnan, China.

Haplotype	Myitsone, Myanmar	Laiza, Myanmar	Yangon, Myanmar^a^	Nu River, China^b^	Xishuangbanna, China^c^
***pvdhfr* haplotype**					
**117N**	5 (5.8%)				
**99S**		55 (36.4%)			6 (11.3%)
**58R/117N**	5 (5.8%)	7 (4.6%)	7 (46.6%)	18 (8.2%)	
**57L/58R**	1(1.2%)				
**57I/58R/61M**		5 (3.3%)			
**58R/99R/117T**		1 (0.7%)			
**58R/99S/117T**		1 (0.7%)			
**57I/58R/61M/117T**	28 (32.6%)	47 (31.1%)		52 (23.6%)	4 (7.5%)
**57L/58R/61M/117T**	38 (44.2%)	19 (12.6%)	3 (20.0%)	80 (36.4%)	28 (52.8%)
**13L/57L/58R/61M/117T**	2 (2.3%)	2 (1.3%)			
**total**	86	151	15	220	53
***pvdhps* haplotype**					
**383G**	17 (17.3%)	44 (31.0%)	3 (20.0%)	26 (12.5%)	13 (24.5%)
**553G**	6 (6.1%)	2 (1.4%)			
**383G/553G**	40 (40.8%)	44 (31.0%)	4 (26.7%)	99 (47.6%)	11 (20.8%)
**383G/571Q**		9 (6.3%)			
**382A/383G**	8 (8.2%)			26 (12.5%)	14 (26.4%)
**382A/383G/553G**	12 (12.2%)	3 (2.1%)		17 (8.2%)	3 (5.7%)
**383G/512M/553G**	2 (2.0%)				
**383G/512T/553G**	3 (3.1%)				
**383G/549D/553G**	1 (1.0%)				
**383G/512E/553G**		1 (0.7%)			
**382C/383G/512E/553G**		4 (2.8%)			
**total**	98	142	15	208	53

^a^data refer to Lu et al., 2010; ^b^data refer to Ding et al., 2013; ^c^data refer to Huang et al., 2014.

Studies in China and Myanmar have found the prevalence of *pvdhfr* and *pvdhps* drug-resistant mutations in *P. vivax* in regions of Yunnan province, China, including the Xishuangbanna prefecture and areas along the Nu River, and these studies identified parasites highly resistant to SP in subtropical China and Yangon, Myanmar from 2010 to 2014 (Lu et al., 2010; Ding et al., 2013; Huang et al., 2014). The quadruple-mutant 57L/58R/61M/117T allele was the most prevalent *pvdhfr* allele in two study sites in the Yunnan province. However, there were no mutations at positions 13 or 173 in the *pvdhfr* gene reported in parasites from the Xishuangbanna. An altered 173 allele was, however, found in 3.18% of samples analyzed from areas along the Nu River. In addition to this, Yunnan province also reported haplotypes with quintuple allelic variants. Our study profiled mutations in marker genes associated with SP resistance among the *P. vivax* from the China-Myanmar border. The quadruple-mutant 57I/58R/61M/117T allele was also prevalent in Myitsone, Myanmar, and in Yunnan, China (Ding et al., 2013; Huang et al., 2014) but absent from our survey of Laiza and from a previous study in Yangon, Myanmar (Lu et al., 2010).

Beneficial mutations reduce variation in physically proximate regions of the chromosome through “genetic hitchhiking” (McCollum et al., 2008; Artimovich et al., 2015). The polymorphism in six microsatellite loci flanking mutant *pvdhfr* (S58R-S117N and the quadruple mutant genotypes 57I/L/58R/61M/117T) was significantly less than that would be expected in wild type genetic backgrounds. The valley of reduced variation spanning *pvdhfr* is resembles that found for samples from Yunnan and central China (Ding et al., 2013). In each, an approximately 100 kb region suggests local genomic variability has been suppressed by a recent selective sweep. These results strengthen the evidence for a relationship between the specific mutant alleles and selection for drug drug-resistant phenotypes.

Furthermore, favorable alleles experience increased linkage disequilibrium (LD), at least until sufficient time has elapsed to enable recombination to distribute alleles randomly among haplotypes in the haploid blood stages of *Plasmodium* parasites. Therefore, the multiple mutations with S117T can serve as a marker for drug resistance at the China-Myanmar Border. Interestingly, 36.4% of Laiza samples bore the single H99S mutation, which has been reported from other studies (Lu et al., 2012; Huang et al., 2014; Wang et al., 2020).

Our results also elucidate the drug pressure exerted by the antifolatesulfadoxine. Previous study has focused on the A383G and A553G in *pvdhps* gene, which are directly related to sulfadoxine resistance (Korsinczky et al., 2004). All of the mutant cases from our study carried at least one of these two mutations. The most prevalent *pvdhps* haplotype we observed was the double mutant 383G/553G allele. There are additional mutant alleles found in Yunnan, including one non-synonymous mutation at codon 512T/M and triple mutant alleles at codons 372, 495, 561. We observed the triple mutant 382A/383G/553G allele and the double mutant 382A/383G allele in Myitsone, Myanmar, and Yunnan, China, but not in Laiza. Nor were these previously observed in Yangon, Myanmar (Lu et al., 2010). We observed the double mutant 383G/571Q only in nine Laiza cases. Besides the common triple mutant 382A/383G/553G allele described earlier, there are other types of triple mutant *pvdhps* alleles in Laiza and Myitsone. They included one case from Laiza with a 383G/512E/553G allele, one from Myitsone with a 383G/549D/553G allele, two from Myitsone with a 383G/512M/553G allele, and three from Myitsone with a 383G/512T/553G allele. Moreover, we observed the quadruple-mutant 382A/383G/512E/553G allele in four Myitsone cases.

SP combination therapy targets two enzymes in the folate-synthesis pathway, dihydropteroate synthase (DHPS) and dihydrofolate reductase (DHFR). The first enzyme in this pathway, GTP-cyclohydrolase 1 (GCH-1), might also respond to drug pressure. Previous work on *P. falciparum* (Kümpornsin et al., 2014) suggested a compensating effect, by copy-number variation (CNV) of *gch-1*, in parasites bearing resistance mutations in *dhps* and *dhfr*. However, such a relationship has not been established in *P. vivax.*Our study, the first to explore such a relationship in *P. vivax*, identified little evidence of copy number variation in *pvgch1*, suggesting that SP-resistant *P. vivax *may harbor alternative mechanisms to secure sufficient folate. Although microsatellite analysis showed that drug resistance genes experience selective pressure, *pvgch1* copy number variation was only observed only in a small number of cases, suggesting that *P. vivax* population might not undergo* pvgch1 *compensation.

The first-line medication for vivax malaria patients in Myanmar is CQ-PQ. Currently, the molecular markers of primaquine resistance are still unknown. And no molecular markers are confirmed for chloroquine resistance in *P*. *vivax*, while *pvmdr1* and *pvcrt-o* have often been queried since these two genes are involved in chloroquine resistance in *P*. *falciparum*, but the possible role of *Pvcrt-o* and *pvmdr1* in chloroquine resistance is controversial. At this area, the *pvmdr1* and *pvcrt-o genes were* reported (Li et al., 2020).The limit of detection of the PCR and sequencing assays used in the study may rule out the possibility of low-frequency mutation isolates. If we want to get the results, deep-sequence needed to be involved in further study.

## Conclusion

We found that most *P. vivax* sampled from symptomatic patients at the China-Myanmar boarder harbor mutations in *pvdhfr* and *pvdhps* genes associated with drug resistance. In this region of intense transmission, which is the source of many cases of vivax malaria introduced to China, *pvdhfr*, a single mutation (S117T) serves as a marker for the presence of multiple mutations. All of the mutant cases from our study carried mutation A383G or A553G in this gene. Parasites throughout the studied region show genetic evidence of undergoing strong drug pressure. The lack of copy number variation of *pvgch1 *suggests that SP-resistant *P. vivax *may harbor alternative mechanisms to secure sufficient folate.

## Data Availability Statement

The datasets presented in this study can be found in online repositories. The names of the repository/repositories and accession number(s) can be found below: GenBank (*pvdhps* gene accession numbers: MZ234760-MZ234999 and *pvdhfr* gene accession numbers: MZ235000-MZ235236).

## Ethics Statement

The studies involving human participants were reviewed and approved by Kunming Medical University. The patients/participants provided their written informed consent to participate in this study.

## Author Contributions

SF, SW, and WZ performed the statistical analysis and wrote the first draft of the manuscript. DZ, YH, YB, YR, YS, HZ, QY, XL, XC, YZ, CL, ZX, YW, FC, PS, and BR wrote sections of the manuscript. ZY contributed to conception and design of the study. ZY organized the database. All authors contributed to the article and approved the submitted version.

## Funding

This study was under the support of the National Science Foundation of China (31860604 and U1802286), Major science and technology projects of Yunnan Province (2018ZF0081) and International Science and Technology Cooperation-Yunnan International Science and Technology Cooperation Base (202003AE140004. Furthermore, XC and YZ were under sponsoring from the Yunnan Applied Basic Research Projects-Union Foundation (2018FE001-190, 2015FB034, respectively). Also, WZ was under the support of the Education Department Fund of Yunnan Province (2019J1184). BR was supported by USDA Project Detection and Control of Foodborne Parasites for Food Safety (8042-32420-007-00D).

## Conflict of Interest

The authors declare that the research was conducted in the absence of any commercial or financial relationships that could be construed as a potential conflict of interest.
