# Supplementary material for: Polymorphism of Antifolate Drug Resistance in Plasmodium vivax From Local Residents and Migrant Workers Returned From the China-Myanmar Border
Source: Front Cell Infect Microbiol. 2021 Jun 24;11:683423. doi: 10.3389/fcimb.2021.683423 (PMC8265503; doi:10.3389/fcimb.2021.683423)
Supplement: Supplementary file 1 [file DataSheet_1.docx]

**Supplementary Table S1. Primers and PCR cycling conditions**

| **Genes** | **Primers** | **Conditions** |
| --- | --- | --- |
| *pv*dhps | 1F: 5′-GGAAGCCATTCGCTCAACTTATAA-3′  1R: 5′-CGTCAGTTTACCCTCCCCGTT-3′  2F: 5′-GATGGCGGTTTATTTGTCGAT-3′  2R: 5′-GCCTCCCCGCTCATCAGTCT-3′ | Primary PCR:  94 ℃ for 5 min,  40 cycles of 94 ℃ for 30 sec,  53 ℃ for 30 sec,  72 ℃ for 30 sec.  Secondary PCR:  94 ℃ for 5 min,  40 cycles of 94 ℃ for 30 sec,  65 ℃ for 30 sec,  72 ℃ for 1 min 30 sec. |
| *pv*dhfr | 1F: 5′-ACCCTTCCATAGGGAGTCCACTT-3′  1R: 5′ -CGCATTGCAGTTCTCCGAA-3′  2F: 5′-CCCCACCACATAACGAAGTAG-3′  2R: 5′-GCCGTTGATCCTCGTGAAG-3′ | Primary PCR:  94 ℃ for 5 min,  40 cycles of 94 ℃ for 30 sec,  53 ℃ for 30 sec,  72 ℃ for 30 sec.  Secondary PCR:  94 ℃ for 5 min,  40 cycles of 94 ℃ for 30 sec,  65 ℃ for 30 sec,  72 ℃ for 1 min 30 sec. |
| *pvaldolase* | F: 5’-GACAGTGCCACCATCCTTACC-3’  R: 5’-CCTTCTCAACATTCTCCTTCTTTCC-3’, | 95 ℃ for 15 min,  40 cycles of 95 ℃ for 10 sec,  64 ℃ for 20 sec,  72 ℃ for 30 sec. |
| *pvgch1* | F: 5’-TCACCACGAGTATGGCTTTG-3’ and R: 5’-CTCCTTCTCCACCCTTTTGA-3’. | 95 ℃ for 15 min,  40 cycles of 95 ℃ for 10 sec,  64 ℃ for 20 sec,  72 ℃ for 30 sec. |

**Supplementary Table S2. Linkage disequilibrium of *pvdhfr* and *pvdhps* genes in** ***P. vivax* from Laiza**

| ***Pvdhfr*** | | | | | | |  | ***Pvdhps*** | | | | | | |
| --- | --- | --- | --- | --- | --- | --- | --- | --- | --- | --- | --- | --- | --- | --- |
| **Site1** | **Site2** | **Dist** | **D** | **D'** | **R** | **Fisher** |  | **Site1** | **Site2** | **Dist** | **D** | **D'** | **R** | **Fisher** |
| 37 | 169 | 132 | -0.005 | -1.000 | -0.084 | 0.545 |  | **1095** | **1106** | 11 | 0.007 | 1.000 | 0.705 | 0.014* |
| 37 | 171 | 134 | 0.007 | 1.000 | 0.120 | 0.232 |  | 1095 | 1144 | 49 | 0.000 | -1.000 | -0.018 | 1.000 |
| 37 | 174 | 137 | -0.006 | -1.000 | -0.106 | 0.500 |  | 1095 | 1145 | 50 | 0.000 | -1.000 | -0.020 | 1.000 |
| 37 | 182 | 145 | 0.007 | 1.000 | 0.120 | 0.232 |  | 1095 | 1146 | 51 | 0.000 | -1.000 | -0.018 | 1.000 |
| 37 | 282 | 245 | 0.000 | -1.000 | -0.009 | 1.000 |  | 1095 | 1148 | 53 | -0.004 | -1.000 | -0.071 | 1.000 |
| 37 | 295 | 258 | -0.005 | -1.000 | -0.089 | 0.530 |  | 1095 | 1206 | 111 | -0.001 | -1.000 | -0.031 | 1.000 |
| 37 | 296 | 259 | -0.005 | -1.000 | -0.090 | 0.527 |  | 1095 | 1534 | 439 | 0.000 | -1.000 | -0.023 | 1.000 |
| **169** | **171** | 2 | 0.178 | 1.000 | 0.749 | 0.000***B |  | 1095 | 1579 | 484 | 0.000 | -1.000 | -0.010 | 1.000 |
| **169** | **174** | 5 | -0.157 | -1.000 | -0.665 | 0.000***B |  | 1095 | 1658 | 563 | -0.005 | -1.000 | -0.094 | 0.525 |
| **169** | **182** | 13 | 0.178 | 1.000 | 0.749 | 0.000***B |  | 1095 | 1711 | 616 | -0.001 | -1.000 | -0.031 | 1.000 |
| 169 | 282 | 113 | -0.002 | -1.000 | -0.059 | 1.000 |  | 1106 | 1144 | 38 | 0.000 | -1.000 | -0.012 | 1.000 |
| **169** | **295** | 126 | -0.128 | -1.000 | -0.556 | 0.000***B |  | 1106 | 1145 | 39 | 0.000 | -1.000 | -0.014 | 1.000 |
| **169** | **296** | 127 | -0.130 | -1.000 | -0.564 | 0.000***B |  | 1106 | 1146 | 40 | 0.000 | -1.000 | -0.012 | 1.000 |
| **171** | **174** | 3 | -0.221 | -1.000 | -0.887 | 0.000***B |  | 1106 | 1148 | 42 | -0.002 | -1.000 | -0.050 | 1.000 |
| **171** | **182** | 11 | 0.250 | 1.000 | 1.000 | 0.000***B |  | 1106 | 1206 | 100 | 0.000 | -1.000 | -0.022 | 1.000 |
| 171 | 282 | 111 | -0.003 | -1.000 | -0.079 | 1.000 |  | 1106 | 1534 | 428 | 0.000 | -1.000 | -0.016 | 1.000 |
| **171** | **295** | 124 | -0.179 | -1.000 | -0.743 | 0.000***B |  | 1106 | 1579 | 473 | 0.000 | -1.000 | -0.007 | 1.000 |
| **171** | **296** | 125 | -0.182 | -1.000 | -0.753 | 0.000***B |  | 1106 | 1658 | 552 | -0.003 | -1.000 | -0.066 | 1.000 |
| **174** | **182** | 8 | -0.221 | -1.000 | -0.887 | 0.000***B |  | 1106 | 1711 | 605 | 0.000 | -1.000 | -0.022 | 1.000 |
| 174 | 282 | 108 | 0.004 | 1.000 | 0.089 | 0.457 |  | 1144 | 1145 | 1 | -0.001 | -1.000 | -0.025 | 1.000 |
| **174** | **295** | 121 | 0.195 | 0.967 | 0.809 | 0.000***B |  | **1144** | **1146** | 2 | 0.021 | 1.000 | 1.000 | 0.000***B |
| **174** | **296** | 122 | 0.192 | 0.935 | 0.794 | 0.000***B |  | 1144 | 1148 | 4 | -0.006 | -1.000 | -0.087 | 0.568 |
| 182 | 282 | 100 | -0.003 | -1.000 | -0.079 | 1.000 |  | 1144 | 1206 | 62 | -0.001 | -1.000 | -0.038 | 1.000 |
| **182** | **295** | 113 | -0.179 | -1.000 | -0.743 | 0.000***B |  | 1144 | 1534 | 390 | -0.001 | -1.000 | -0.028 | 1.000 |
| **182** | **296** | 114 | -0.182 | -1.000 | -0.753 | 0.000***B |  | 1144 | 1579 | 435 | 0.000 | -1.000 | -0.012 | 1.000 |
| 282 | 295 | 13 | 0.004 | 1.000 | 0.106 | 0.371 |  | 1144 | 1658 | 514 | 0.013 | 1.000 | 0.188 | 0.053 |
| 282 | 296 | 14 | 0.004 | 1.000 | 0.105 | 0.377 |  | 1144 | 1711 | 567 | -0.001 | -1.000 | -0.038 | 1.000 |
| **295** | **296** | 1 | 0.231 | 1.000 | 0.986 | 0.000***B |  | 1145 | 1146 | 1 | -0.001 | -1.000 | -0.025 | 1.000 |
|  |  |  |  |  |  |  |  | 1145 | 1148 | 3 | -0.007 | -1.000 | -0.101 | 0.573 |
|  |  |  |  |  |  |  |  | 1145 | 1206 | 61 | -0.002 | -1.000 | -0.044 | 1.000 |
|  |  |  |  |  |  |  |  | **1145** | **1534** | 389 | 0.027 | 1.000 | 0.891 | 0.000***B |
|  |  |  |  |  |  |  |  | 1145 | 1579 | 434 | 0.000 | -1.000 | -0.014 | 1.000 |
|  |  |  |  |  |  |  |  | **1145** | **1658** | 513 | 0.017 | 1.000 | 0.217 | 0.019* |
|  |  |  |  |  |  |  |  | 1145 | 1711 | 566 | -0.002 | -1.000 | -0.044 | 1.000 |
|  |  |  |  |  |  |  |  | 1146 | 1148 | 2 | -0.006 | -1.000 | -0.087 | 0.568 |
|  |  |  |  |  |  |  |  | 1146 | 1206 | 60 | -0.001 | -1.000 | -0.038 | 1.000 |
|  |  |  |  |  |  |  |  | 1146 | 1534 | 388 | -0.001 | -1.000 | -0.028 | 1.000 |
|  |  |  |  |  |  |  |  | 1146 | 1579 | 433 | 0.000 | -1.000 | -0.012 | 1.000 |
|  |  |  |  |  |  |  |  | 1146 | 1658 | 512 | 0.013 | 1.000 | 0.188 | 0.053 |
|  |  |  |  |  |  |  |  | 1146 | 1711 | 565 | -0.001 | -1.000 | -0.038 | 1.000 |
|  |  |  |  |  |  |  |  | 1148 | 1206 | 58 | -0.017 | -1.000 | -0.154 | 0.112 |
|  |  |  |  |  |  |  |  | 1148 | 1534 | 386 | -0.009 | -1.000 | -0.113 | 0.327 |
|  |  |  |  |  |  |  |  | 1148 | 1579 | 431 | 0.005 | 1.000 | 0.142 | 0.261 |
|  |  |  |  |  |  |  |  | **1148** | **1658** | 510 | -0.085 | -0.858 | -0.399 | 0.000***B |
|  |  |  |  |  |  |  |  | 1148 | 1711 | 563 | -0.017 | -1.000 | -0.154 | 0.112 |
|  |  |  |  |  |  |  |  | 1206 | 1534 | 328 | -0.002 | -1.000 | -0.050 | 1.000 |
|  |  |  |  |  |  |  |  | 1206 | 1579 | 373 | 0.000 | -1.000 | -0.022 | 1.000 |
|  |  |  |  |  |  |  |  | **1206** | **1658** | 452 | -0.024 | -1.000 | -0.204 | 0.013* |
|  |  |  |  |  |  |  |  | **1206** | **1711** | 505 | 0.059 | 1.000 | 1.000 | 0.000***B |
|  |  |  |  |  |  |  |  | 1534 | 1579 | 45 | 0.000 | -1.000 | -0.016 | 1.000 |
|  |  |  |  |  |  |  |  | **1534** | **1658** | 124 | 0.022 | 1.000 | 0.244 | 0.007** |
|  |  |  |  |  |  |  |  | 1534 | 1711 | 177 | -0.002 | -1.000 | -0.050 | 1.000 |
|  |  |  |  |  |  |  |  | 1579 | 1658 | 79 | -0.003 | -1.000 | -0.066 | 1.000 |
|  |  |  |  |  |  |  |  | 1579 | 1711 | 132 | 0.000 | -1.000 | -0.022 | 1.000 |
|  |  |  |  |  |  |  |  | **1658** | **1711** | 53 | -0.024 | -1.000 | -0.204 | 0.013* |

Note: * 0.01<P<0.05; ** 0.001<P<0.01; *** P<0.001; B, significant by the Bonferroni procedure

**Supplementary Table S3． Linkage disequilibrium of *pvdhfr* and *pvdhps* genes in** ***P. vivax* from Myitsone**

| ***Pvdhfr*** | | | | | | |  | ***Pvdhps*** | | | | | | |
| --- | --- | --- | --- | --- | --- | --- | --- | --- | --- | --- | --- | --- | --- | --- |
| **Site1** | **Site2** | **Dist** | **D** | **D'** | **R** | **Fisher** |  | **Site1** | **Site2** | **Dist** | **D** | **D'** | **R** | **Fisher** |
| 37 | 169 | 132 | -0.008 | -1.000 | -0.107 | 0.556 |  | **1144** | **1146** | 2 | 0.162 | 1.000 | 1.000 | 0.000***B |
| 37 | 171 | 134 | -0.005 | -1.000 | -0.077 | 1.000 |  | 1144 | 1148 | 4 | -0.031 | -1.000 | -0.215 | 0.036* |
| 37 | 174 | 137 | -0.003 | -1.000 | -0.062 | 1.000 |  | 1144 | 1485 | 341 | -0.002 | -1.000 | -0.051 | 1.000 |
| 37 | 182 | 145 | -0.005 | -1.000 | -0.079 | 1.000 |  | 1144 | 1646 | 502 | -0.002 | -1.000 | -0.051 | 1.000 |
| 37 | 267 | 230 | 0.000 | -1.000 | -0.017 | 1.000 |  | 1144 | 1658 | 514 | 0.011 | 0.081 | 0.056 | 0.605 |
| 37 | 295 | 258 | 0.000 | -1.000 | -0.017 | 1.000 |  | **1146** | **1148** | 2 | -0.031 | -1.000 | -0.215 | 0.036* |
| 37 | 296 | 259 | 0.000 | -1.000 | -0.017 | 1.000 |  | 1146 | 1485 | 339 | -0.002 | -1.000 | -0.051 | 1.000 |
| **169** | **171** | 2 | -0.064 | -1.000 | -0.345 | 0.001***B |  | 1146 | 1646 | 500 | -0.002 | -1.000 | -0.051 | 1.000 |
| **169** | **174** | 5 | -0.045 | -1.000 | -0.280 | 0.007** |  | 1146 | 1658 | 512 | 0.011 | 0.081 | 0.056 | 0.605 |
| **169** | **182** | 13 | -0.068 | -1.000 | -0.357 | 0.000***B |  | 1148 | 1485 | 337 | -0.002 | -1.000 | -0.043 | 1.000 |
| 169 | 267 | 98 | -0.004 | -1.000 | -0.075 | 1.000 |  | 1148 | 1646 | 498 | -0.002 | -1.000 | -0.043 | 1.000 |
| 169 | 295 | 126 | -0.004 | -1.000 | -0.075 | 1.000 |  | **1148** | **1658** | 510 | 0.039 | 0.388 | 0.226 | 0.038* |
| 169 | 296 | 127 | -0.004 | -1.000 | -0.075 | 1.000 |  | 1485 | 1646 | 161 | 0.000 | -1.000 | -0.010 | 1.000 |
| **171** | **174** | 3 | 0.112 | 1.000 | 0.811 | 0.000***B |  | 1485 | 1658 | 173 | -0.004 | -1.000 | -0.074 | 1.000 |
| **171** | **182** | 11 | 0.156 | 1.000 | 0.965 | 0.000***B |  | 1646 | 1658 | 12 | -0.004 | -1.000 | -0.074 | 1.000 |
| 171 | 267 | 96 | -0.002 | -1.000 | -0.054 | 1.000 |  |  |  |  |  |  |  |  |
| 171 | 295 | 124 | 0.009 | 1.000 | 0.219 | 0.198 |  |  |  |  |  |  |  |  |
| 171 | 296 | 125 | 0.009 | 1.000 | 0.219 | 0.198 |  |  |  |  |  |  |  |  |
| **174** | **182** | 8 | 0.110 | 1.000 | 0.783 | 0.000***B |  |  |  |  |  |  |  |  |
| 174 | 267 | 93 | -0.002 | -1.000 | -0.044 | 1.000 |  |  |  |  |  |  |  |  |
| 174 | 295 | 121 | 0.010 | 1.000 | 0.269 | 0.140 |  |  |  |  |  |  |  |  |
| 174 | 296 | 122 | 0.010 | 1.000 | 0.269 | 0.140 |  |  |  |  |  |  |  |  |
| 182 | 267 | 85 | -0.002 | -1.000 | -0.056 | 1.000 |  |  |  |  |  |  |  |  |
| 182 | 295 | 113 | 0.009 | 1.000 | 0.211 | 0.209 |  |  |  |  |  |  |  |  |
| 182 | 296 | 114 | 0.009 | 1.000 | 0.211 | 0.209 |  |  |  |  |  |  |  |  |
| 267 | 295 | 28 | 0.000 | -1.000 | -0.012 | 1.000 |  |  |  |  |  |  |  |  |
| 267 | 296 | 29 | 0.000 | -1.000 | -0.012 | 1.000 |  |  |  |  |  |  |  |  |
| **295** | **296** | 1 | 0.011 | 1.000 | 1.000 | 0.012* |  |  |  |  |  |  |  |  |

Note: * 0.01<P<0.05; ** 0.001<P<0.01; *** P<0.001; B, significant by the Bonferroni procedure

**Supplementary Table S4． P-values for the observed heterozygosity from Wilcoxon Signed-Rank Test of the mutation–drift equilibrium estimated for the six microsatellite loci**

|  | IAM model | | | SMM model | | |
| --- | --- | --- | --- | --- | --- | --- |
|  | H _deficiency_ | H _excess_ | H _excess and deficiency_ | H _deficiency_ | H _excess_ | H _excess and deficiency_ |
| Myitsone |  |  |  |  |  |  |
| WILD-TYPE | 0.4062 | 0.6875 | 0.8125 | 0.4062 | 0.6875 | 0.8125 |
| S58R-S117N | 0.9922 | 0.0156* | 0.0313* | 0.9922 | 0.0156* | 0.0313* |
| 57L-58R-61M-117T | 0.3438 | 0.7188 | 0.6875 | 0.0078 | 1.0000 | 0.0156 |
| 57I-58R-61M-117T | 0.2813 | 0.7813 | 0.5625 | 0.0391 | 0.9766 | 0.0781 |
| Laiza |  |  |  |  |  |  |
| WILD-TYPE | 0.5000 | 0.5781 | 1.0000 | 0.0547 | 0.9609 | 0.1093 |
| S58R-S117N | 0.8750 | 0.1875 | 0.3750 | 0.6250 | 0.8125 | 1.0000 |
| 57L-58R-61M-117T | 0.6563 | 0.4219 | 0.8438 | 0.0547 | 0.9609 | 0.1094 |

***P* < 0.05**
