# Supplementary material for: Polymorphism of Antifolate Drug Resistance in Plasmodium vivax From Local Residents and Migrant Workers Returned From the China-Myanmar Border
Source: Front Cell Infect Microbiol. 2021 Jun 24;11:683423. doi: 10.3389/fcimb.2021.683423 (PMC8265503; doi:10.3389/fcimb.2021.683423)
Supplement: Supplementary file 2 [file Presentation_1.pptx]

## Slide 1
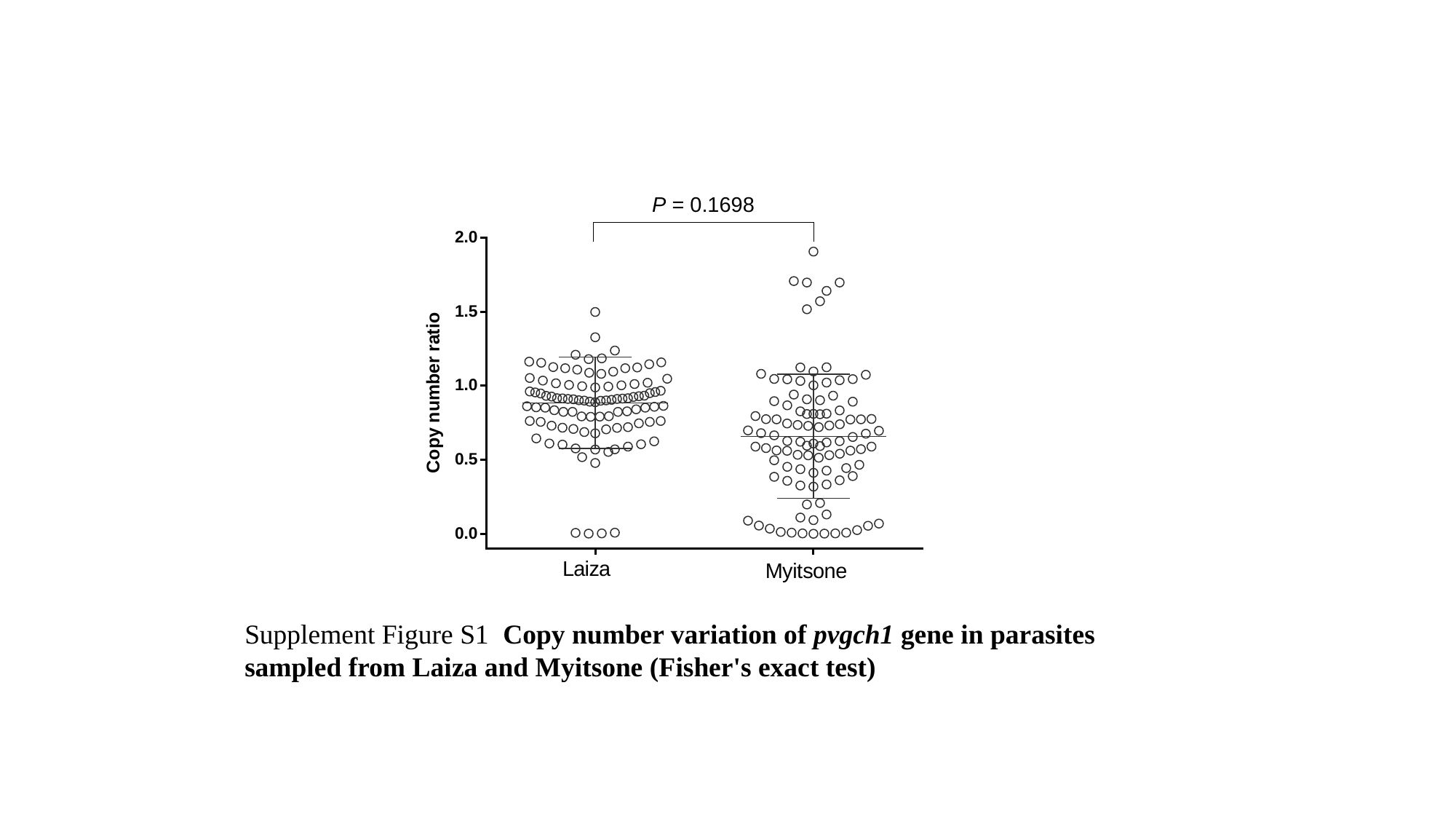

| |
| --- |
Supplement Figure S1 Copy number variation of pvgch1 gene in parasites sampled from Laiza and Myitsone (Fisher's exact test)
